# How aging affects visuomotor adaptation and retention in a precision walking paradigm

**DOI:** 10.1038/s41598-020-80916-8

**Published:** 2021-01-12

**Authors:** Amanda Bakkum, Shaila M. Gunn, Daniel S. Marigold

**Affiliations:** Department of Biomedical Physiology and Kinesiology, Simon Fraser University, 8888 University Drive, Burnaby, BC V5A 1S6 Canada

**Keywords:** Motor control, Learning and memory

## Abstract

Motor learning is a lifelong process. However, age-related changes to musculoskeletal and sensory systems alter the relationship (or mapping) between sensory input and motor output, and thus potentially affect motor learning. Here we asked whether age affects the ability to adapt to and retain a novel visuomotor mapping learned during overground walking. We divided participants into one of three groups (n = 12 each) based on chronological age: a younger-aged group (20–39 years old); a middle-aged group (40–59 years old); and an older-aged group (60–80 years old). Participants learned a new visuomotor mapping, induced by prism lenses, during a precision walking task. We assessed retention one-week later. We did not detect significant effects of age on measures of adaptation or savings (defined as faster relearning). However, we found that older adults demonstrated reduced initial recall of the mapping, reflected by greater foot-placement error during the first adaptation trial one-week later. Additionally, we found that increased age significantly associated with reduced initial recall. Overall, our results suggest that aging does not impair adaptation and that older adults can demonstrate visuomotor savings. However, older adults require some initial context during relearning to recall the appropriate mapping.

## Introduction

Age-related physiological changes to musculoskeletal and sensory systems can alter the relationship (or mapping) between sensory input and motor output. The ability to recalibrate movement in response to these sensorimotor changes, and retain what is learned, is essential for successful motor performance over a lifetime. Failure to maintain accurate sensorimotor mappings may exacerbate age-related mobility issues and increase the risk of falls—a leading cause of injury-related deaths for older adults^1^. As the world’s population ages, we need a deeper understanding of how the healthy aging process influences sensorimotor adaptation and retention.

Both younger and older adults are able to adapt movement in response to sensorimotor perturbations. Most research in this area has focused on adaptation during upper limb movements, such as reaching or throwing to targets^2–5^, though a few recent studies have also explored adaptation during locomotion^6–8^. Aging may impair motor learning, reflected by slower adaptation^3,4,6,8–11^ and reduced overall adaptation levels^2,11–13^. For example, Fernández-Ruiz et al.^3^ showed that older adults are slower to adapt their throwing trajectories in response to visual perturbations compared to their younger counterparts. In contrast, other studies did not detect differences in motor adaptation between younger and older adults^4,7,14,15^. These conflicting findings are evident across a variety of adaptation protocols, including force field, visuomotor rotation, prismatic, and split-belt treadmill paradigms. Thus, despite the growing number of studies in this area, the extent to which aging affects sensorimotor adaptation is unclear.

Savings, or faster relearning, is often regarded as an indicator of long-term motor memory (or retention). Research on the effects of healthy aging on motor memory retention is scarce, though some studies show reduced savings with advanced age^7,8,16^. For example, Malone and Bastian^7^ showed that the healthy aging process weakened motor memories formed during split-belt treadmill walking, reflected by decreased savings of previously learned step symmetry following five-minute rest periods. Similarly, Sombric et al.^8^ showed that older adults exhibited naïve-like behavior, or reduced savings, after repeated exposure to a split-belt treadmill locomotor task. However, these age-related deficits to savings are not always apparent^5,13^. A common feature across these studies is that savings was assessed over short periods of time (less than 24 h), often within the same testing session as adaptation. In previous studies of healthy young adults, we demonstrated that the motor memory acquired after learning a novel visuomotor mapping in a precision walking paradigm is retained for at least 1 year^17,18^. However, the effects of aging on motor memory retention are still unknown.

In this study, we asked whether age affects the ability to adapt to and retain a novel visuomotor mapping learned during overground walking. We divided participants into three different age groups and had them adapt to a novel visuomotor mapping induced by prism lenses while performing a precision walking task. The prism lenses altered the relationship between visual input and motor output, causing a mismatch between what the participants saw and how they moved. To regain movement accuracy and perform the task successfully, participants had to learn the new visuomotor mapping. We then tested retention of this new mapping by having participants repeat the adaptation protocol one-week later.

## Methods

### Participants

We collected data from fifteen new participants and combined them with data from twenty-one participants used in one of two previous studies^17,19^. Thus, a total of thirty-six participants with no known musculoskeletal, neurological, or visual disease were included in this study. We divided participants into one of three adaptation groups based on their chronological age (n = 12 each). For the younger-aged group, participants were between 20 and 39 years old (mean ± SD: 28.3 ± 6.9 years; 5 females, 7 males). Participants in the middle-aged group were between 40 and 59 years old (50.3 ± 6.1 years; 8 females, 4 males). Finally, participants in our older-aged group were between the ages of 60 and 80 years old (69.0 ± 5.4 years; 4 females, 8 males). We excluded data from one participant in the older adult group because they showed no evidence of adaptation, it is unclear whether they understood the task instructions, and their data were significant outliers based on the distribution of studentized residuals (i.e., studentized residuals greater than 3.5).

The Office of Research Ethics at Simon Fraser University approved the study protocol, and we conducted all experiments in accordance with the relevant guidelines and regulations. All participants provided informed, written consent prior to their participation.

### Experimental task and protocol

**Figure 1:**
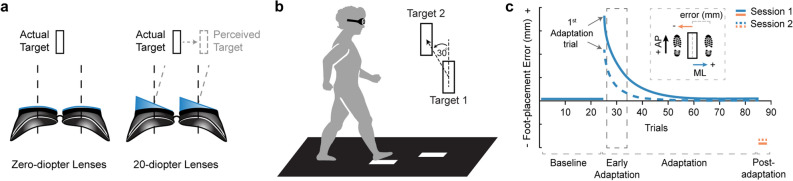
Experimental setup and protocol. (**a**) A simulated view of a target through the goggles coupled with zero-diopter (non-visual-field-shifting) lenses and 20-diopter prism lenses that shift the perceived location of the target 11.4° to the right. (**b**) A schematic of the visually guided precision walking task. Participants walked and stepped with the right and left foot onto the center on two sequential targets on the ground. (**c**) An illustration of the predicted end-point error profiles for each phase of testing. All participants performed baseline, adaptation, and post-adaptation phases. *Inset:* Positive ( +) and negative ( −) medial–lateral (ML) foot-placement error. AP = anterior–posterior direction in motion capture space.

Participants learned a novel visuomotor mapping induced by prism lenses (Fig. 1a) while performing a precision walking task (Fig. 1b). This task involved walking along a 6 m long path and stepping with the right and left foot onto the center on two sequential targets (15 × 30 cm) without stopping^17,19^. We placed the first target in the same position for all participants. We then positioned the second target at a 30° counterclockwise angle with respect to the plane of progression and at a distance of ~ 90% of the height (from the floor) of the participant’s greater trochanter. Participants took a minimum of two steps before and after the step to the targets. To track movement, we recorded (at 100 Hz) infrared-emitting position markers placed on the participant’s chest (in line with the sternum) and bilaterally on each mid-foot (second-third metatarsal head) with an Optotrak Certus motion capture camera (Northern Digital, Waterloo, ON, Canada).

We determined how age affected initial adaptation to the novel visuomotor mapping and relearning one week later. Participants performed baseline, adaptation, and post-adaptation phases while wearing goggles fitted with 20-diopter prism lenses or zero-diopter (non-visual-field-shifting) lenses (Fig. 1a). The goggles were designed to block a portion of the peripheral visual field so that participants had to look through the lenses to perform the task. During the first testing session, participants performed 25 baseline trials while wearing zero-diopter lenses. Participants then performed 60 adaptation trials while wearing the 20-diopter prism lenses. Finally, participants performed a single trial with the zero-diopter lenses to determine if they had updated their internal model (i.e., post-adaptation trial). To assess relearning, participants returned one week later and repeated the adaptation and post-adaptation phases.

For the first trial of each phase, participants started at a distance of 1.8 m from the first target. Thereafter, we randomized the participant’s anterior–posterior (AP) starting location (between 1.5—2.5 m) for each trial to prevent participants from learning a specific walking sequence and increase the demand for visual feedback. We instructed participants to step accurately onto the medial–lateral (ML) center of each target and to perform the task at a quick and constant pace to reduce the possibility of making rapid online corrections of leg/foot trajectory during the steps to targets. To minimize adaptation between trials, participants had their eyes open only when they were performing the task. To begin a trial, participants opened their eyes once cued by a verbal command and immediately started walking to the targets. An experimenter demonstrated the task prior to the testing protocol and helped guide the participant back to the start of the walkway between each trial.

### Data and statistical analysis

We used kinematic data (filtered using a fourth-order, low-pass Butterworth algorithm with a cut-off frequency of 6 Hz) to calculate foot placement on targets, defined as the moment the mid-foot marker’s AP velocity and acceleration profiles stabilized to zero^17,19^. We defined ML foot-placement error as the ML distance between the mid-foot marker and the center of the target, which served to quantify adaptation across trials and visuomotor savings between testing sessions. A positive value represents errors in the direction of the prism shift (i.e., to the right), and a negative value represents errors in the left direction, opposite to the prism shift (Fig. 1c). We analyzed each target separately, as previous work shows that the legs adapt and generalize differently depending on the target sequence^20,21^, and the second target step may be biased by the first. Thus, we focused on and report the results of the step to the first target. We verified the absence of sudden changes in foot marker trajectory to confirm that all participants performed the precision walking task without making online corrections of the step to the target.

For all statistical analyses, we used JMP software, Version 15 (Copyright 2019 SAS Institute Inc. SAS and all other SAS Institute Inc. product or service names are registered trademarks or trademarks of the SAS Institute Inc. Cary, NC) with an alpha level of 0.05. For ANOVA’s, we included participant as a random effect and used Tukey’s post hoc tests as necessary. To determine the effects of aging on adaptation, we compared foot-placement error during the baseline phase (average of the last ten trials), first adaptation trial, late adaptation (average of the last ten trials), and post-adaptation trials during the first testing session using a two-way (Group x Phase) mixed-model ANOVA. When checking whether the assumptions of an ANOVA were met, we found three potential data outliers based on large studentized residuals. We subsequently excluded these data for this analysis.

To assess relearning of the mapping one week later, we used two measures: the first adaptation trial error (representing the initial recall of the mapping) and early adaptation error (i.e., mean of adaptation trials 2 – 8). Early adaptation error captures the large, rapid reduction in error early in the adaptation phase as a means to capture savings (see Fig. 1c) and is similar to methods used by others^17,22,23^. We then performed separate two-way (Group x Session) mixed-model ANOVAs.

To further determine how age relates to initial recall, we used a linear regression model with age as the regressor and the difference in first adaptation trial foot-placement error between testing sessions as the response variable. To further determine how age relates to savings, we used a linear regression model with age as the regressor and the difference in early adaptation foot-placement error between testing sessions as the response variable.

## Results

### Older adults can adapt to a novel visuomotor mapping during overground locomotion

**Figure 2:**
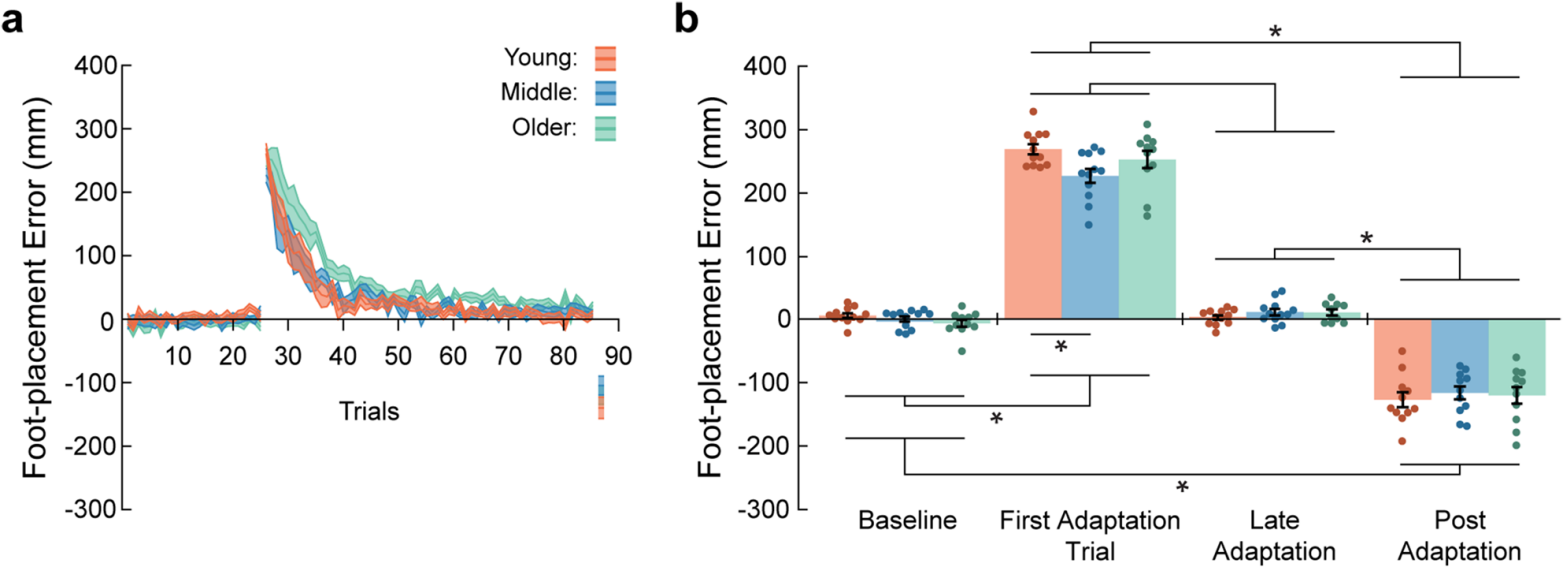
Aging and visuomotor adaptation. (**a**) Group mean ± SE foot-placement error across all trials for baseline, adaptation, and post-adaptation phases during the first testing session. (**b**) Group mean ± SE foot-placement error for the baseline phase (average of the last ten trials), first adaptation trial, late adaptation (average of the last ten trials), and post-adaptation trials for the first target during the first testing session. *Indicate that values are significantly different from each other (*p* < 0.05).

All participants demonstrated a large, rightward deviation in foot placement to the first target upon initial exposure to the 20-diopter prism lenses. As participants adapted to the prisms, foot-placement error gradually returned to near-baseline levels. Subsequently, removal of the prism lenses during post-adaptation resulted in a large foot-placement error to left (i.e., a negative aftereffect). These results are illustrated in Fig. 2.

To determine the effects of aging on adaptation, we compared foot-placement error over several phases during the first testing session. We found a significant Group x Phase interaction (Fig. 2b; F_6,94_ = 2.43, *p* = 0.031). Post hoc tests indicated significantly greater foot-placement error in the first adaptation trial compared to the other phases. Furthermore, foot-placement error for the post adaptation trials differed significantly from the other phases. However, we did not detect significant differences between age groups for each phase, with the exception of the first adaptation trial. In this case, the middle age group demonstrated significantly less initial error compared to the younger age group. Overall, these results suggest that aging did not impair adaptation to the novel, prism-induced visuomotor mapping.

### Older adults demonstrate reduced initial recall of the learned visuomotor mapping

**Figure 3:**
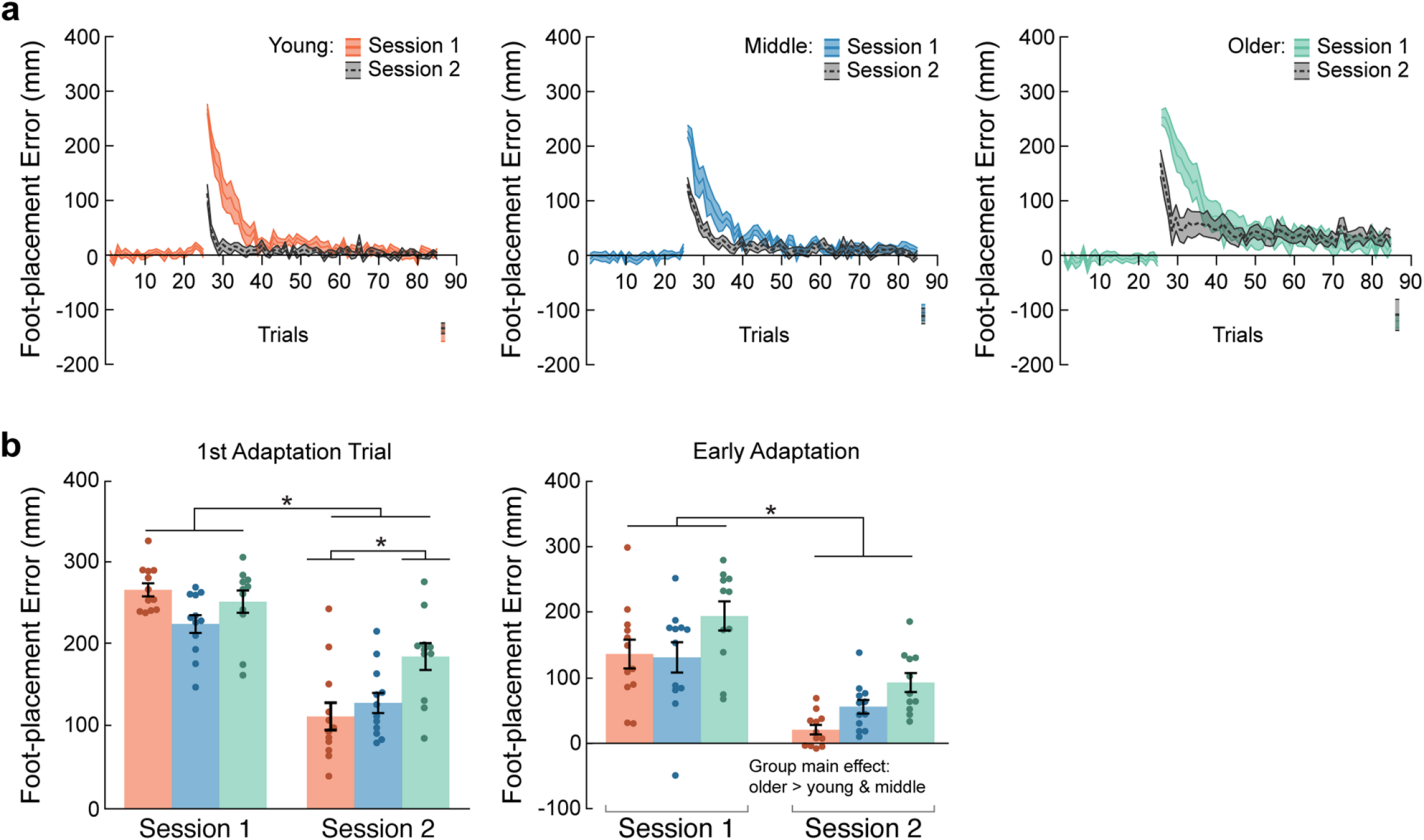
Aging and visuomotor retention. (**a**) Group mean ± SE foot-placement error for all trials in the baseline, adaptation, and post-adaptation phases across testing sessions. (**b**) Group mean ± SE foot-placement error for the first adaptation and early adaptation trials of the first target across testing sessions. Testing sessions occurred one week apart. *Indicate that values are significantly different from each other (*p* < 0.05).

To assess initial recall and savings, participants repeated the adaptation protocol one week later. Figure 3a illustrates group mean foot-placement error across trials for both testing sessions. Error is clearly reduced during the second testing session in all groups. Initially, we compared the first adaptation trial foot-placement error between testing sessions (Fig. 3b). We found a significant Group x Session interaction for this measure (F_2,32_ = 6.7, *p* = 0.004). All groups demonstrated smaller first adaptation trial error during the second compared to first testing session. However, older adults had greater first adaptation trial error than young adults during the second testing session. Next, to quantify savings, we compared early adaptation error between testing sessions (Fig. 3b). All groups had reduced early adaptation error during the second testing session compared to first testing session (Session main effect: F_1,32_ = 55.6, *p* < 0.0001). In addition, the older adult group demonstrated greater early adaptation error than the young and middle age groups (Group main effect: F_2,32_ = 6.1, *p* = 0.006).

**Figure 4:**
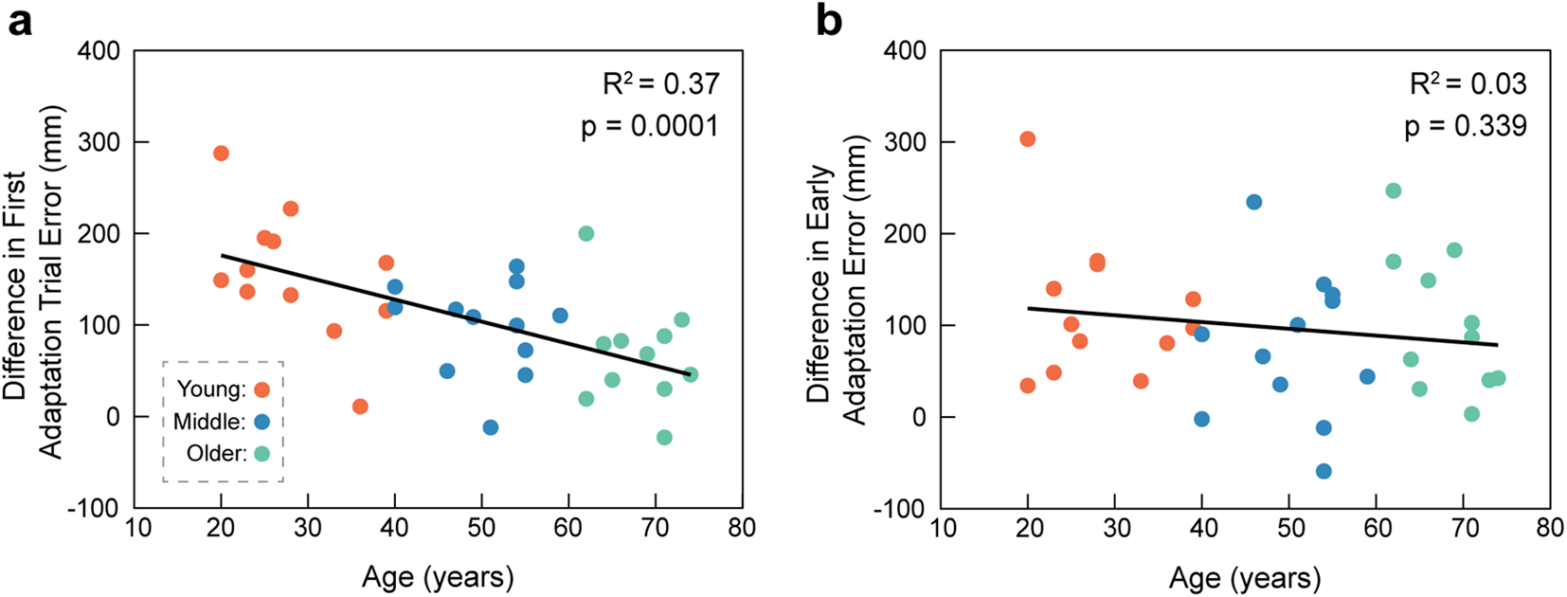
Relationship between aging and initial recall or savings. (**a**) Scatter plot illustrating the relationship between age and the difference in first adaptation trial foot-placement error between testing sessions. (**b**) Scatter plot illustrating the relationship between age and the difference in early adaptation foot-placement error between testing sessions. Testing sessions occurred one week apart. The solid lines show the linear fits obtained from the regression analyses.

Given the age-group effect with the first adaptation trial error in the second testing session, we asked whether there was an association between age and the initial recall of the prism-induced mapping one-week later. To address this question, we used a linear regression model with age as the regressor and the difference in first adaptation trial foot-placement error between testing sessions as the response variable. Figure 4a shows the scatter plot of these data. We found that increased age significantly associated with reduced initial recall (R^2^ = 0.37, coefficient = -2.41, *p* = 0.0001), reflected by a smaller difference in foot-placement error on the first adaptation trial between sessions. We next asked whether there was an association between age and savings of the prism-induced mapping one-week later. To address this question, we used a linear regression model with age as the regressor and the difference in early adaptation foot-placement error between testing sessions as the response variable. Figure 4b shows the scatter plot of these data. However, we did not detect a significant relationship (R^2^ = 0.03, coefficient = -0.74, *p* = 0.339).

## Discussion

People must maintain accurate sensorimotor mappings to safely interact with the environment despite age-related changes in visual and motor function. Here we asked whether age affects the ability to adapt to and retain a novel visuomotor mapping learned during overground walking. Our findings show that aging did not impair visuomotor adaptation and that older adults demonstrate visuomotor savings. However, we found that increased age significantly associated with reduced initial recall of the mapping one week later. These results suggest that, while older adults demonstrate long-term motor memory retention, they might require some initial context during relearning—i.e., exposure to the previously learned mapping and its effect—to recall the appropriate mapping.

### The effects of aging on visuomotor adaptation and retention during overground walking

Our results show that older adults reduced their foot-placement error similarly to middle-aged and younger adults as they adapted to the novel visuomotor mapping. Ours is not the first study to show this with sensorimotor adaptation^4,7,14,15^, though our results contrast previous findings that demonstrate age-related deficits^2–4,6,8–11^. One study in particular showed that older adults were slower to adapt to a new visuomotor mapping during locomotion^6^. The reasons for this discrepancy are unclear, though they may relate to differences between the adaptation protocols and walking tasks being evaluated. For example, our study assessed adaptation during a precision walking task using foot-placement error, where participants had to make a single precise step with each foot to the centre of a target as they walked. In contrast, the other study measured the extent of lateral deviation along a path, where participants could continually make adjustments to their walking trajectory. Thus, the nature and precision demand of the two tasks differ. Furthermore, our study assessed adaptation to a 20-diopter visuomotor perturbation (~ 11.4º), whereas Nemanich and Earhart^6^ used 30-diopter lenses that induce a larger visual field shift (~ 17.1º). Previous research demonstrates that error size can affect sensorimotor adaptation^24–26^. It is possible that the effects of aging on sensorimotor adaptation are more pronounced with larger visuomotor perturbations. We also show that aging did not impair storage of the new mapping in that all groups demonstrated similar negative aftereffects during the post-adaptation phase. These results support findings from several studies that show similar magnitudes of aftereffects between age groups^2,4,6,12,15,27,28^. Overall, we found that aging did not impair the ability to adapt to a novel visuomotor mapping during overground walking.

Sensorimotor adaptation can induce long-term behavioural changes whereby people demonstrate savings (or faster adaptation) when they re-encounter a familiar perturbation. This retention of motor memories is evident in both reaching^29–31^ and walking studies^22,32–34^, and can persist for extended periods of time. For instance, previous research from our lab demonstrates that visuomotor memories formed during overground locomotion are stored for at least one week and even up to one year, highlighting the robustness of visuomotor memories associated with walking^17–19^. Consistent with this work, we found that all participants reduced their foot-placement error and adapted faster when exposed to the novel visuomotor mapping a second time, one week later. Although two studies have shown short-term retention with aging^5,13^, to our knowledge, this is the first study to demonstrate longer-term retention of sensorimotor adaptation in older adults.

### The effects of aging on implicit and explicit learning processes

Visuomotor adaptation can be achieved through cerebellum-dependent, internal model recalibration driven by sensory predictions errors^35–39^, and/or through explicit, strategic control^36,40–42^. Similarly, research shows that savings may result from a memory of errors experienced during adaptation^43^, or through the rapid recall of a deliberate aiming strategy^44,45^. Recent studies have linked age-related deficits in sensorimotor adaptation with deterioration in explicit learning processes^5,16,46^. For example, older adults demonstrate impaired performance on motor tasks that engage explicit strategies, suggesting that the cognitive components of sensorimotor adaptation are reduced with increased age^5^. In contrast, implicit learning processes appear spared with aging^2,4,16,47^. Thus, one explanation for the inconsistencies in the literature may be linked to the nature of experimental paradigms used to assess adaptation and the degree to which they engage explicit learning processes.

Although we did not directly measure implicit and explicit learning, evidence suggests that both processes contribute to adaptation during our task. For example, performance during prism adaptation is thought to reflect a rapid, strategic control process aimed at minimizing early performance errors and a slower spatial realignment, or recalibration, process that is evident from aftereffects^48^. Indeed, our previous work demonstrated implicit, model-based learning with prism adaptation during a precision walking task similar to that used in this study^23^. In the present study, we found that older adults exhibited similar aftereffects following adaptation, which is indicative of spatial realignment and also supports the idea that internal model recalibration remains intact with aging. However, while our older age group demonstrated visuomotor savings, they had reduced initial recall of the mapping one week later. Furthermore, we showed that increasing age significantly associated with reduced initial recall (see Fig. 4). These findings may align with studies that show deterioration of explicit memory systems with age^5,16,46^. One study in particular suggests that age-related deficits in sensorimotor adaptation are not due to impaired retention of motor memory, but rather a reduction in the cognitive component of learning^5^. It is possible that reductions in explicit memory affected the older adult’s ability to recall a deliberate aiming strategy early in relearning. However, once the older adults experienced a familiar error during the first relearning trial, they were able to select the appropriate mapping and demonstrate savings. This observation is consistent with previous studies showing that older adults have difficulty switching motor behaviors across different contexts, such as transitioning between split-belt treadmill and overground walking^8,49^. Furthermore, while it may be faster and more advantageous to recall context-specific motor memories^50^, these results support recent evidence that savings in sensorimotor adaptation does not depend exclusively on the ability to recall an explicit strategy^51^. Taken together, age-related cognitive deficits may impair the ability to recall an explicit strategy. However, experience with a previously learned error is sufficient to set the context to recall the appropriate mapping and demonstrate savings.

### The effects of aging on the neural structures involved in visuomotor adaptation and retention

The effects of aging on the brain are widespread, which may have multiple implications for motor learning. The cerebellum, for example, is essential for internal model recalibration and is implicated in visuomotor adaptation^39,52–56^. The cerebellum also plays a critical role in both the formation and retention of motor memories^57,58^. With increased age, however, we see large reductions in grey matter volume, or shrinking, of the cerebellum^59^. Age-related cerebellar degeneration is thought to contribute to the impaired sensorimotor adaptation in some older adult studies^11,60–62^. More recently, however, a study from Vandervoorde and Orban de Xivry^5^ demonstrated that internal model recalibration does not deteriorate with age despite degeneration to the cerebellum. Instead, these authors suggest that age-related deficits in sensorimotor adaptation result from reductions in the cognitive components of motor learning. Likewise, Wolpe et al.^46^ found that decreased sensorimotor adaptation with aging was associated with reduced gray matter volume in regions linked to explicit learning (e.g., the striatum and prefrontal cortex) and not the cerebellum. Finally, a recent study found that activation in brain regions related to cognitive processes, including striatal, parietal, and cingulate cortical areas positively correlated with sensorimotor savings^63^. Interestingly, the striatum is thought to function as an adaptive search mechanism that retrieves the appropriate sensorimotor representations for a given environment^64^. Thus, it is possible that age-related volume loss to this region may contribute to the reduced initial recall we observed in our older adults. Taken together, these findings converge with the results from behavioural studies demonstrating that age-related deficits in sensorimotor adaptation are driven by a decline in explicit learning processes. However, the impact of age-related brain changes on motor learning is still unclear and further research is warranted.

## Conclusions

This study extends the research on short-term motor memory retention with aging by demonstrating that older adults are able to retain a learned visuomotor mapping for an extended period of time. Furthermore, this work contributes to the growing literature that suggests age-related deficits to motor learning may be driven by a reduction in explicit learning processes. Future research should determine if explicit learning components can be leveraged to facilitate long-term motor memory retention in older adults, which is important for designing rehabilitation programs for an aging population.

## Data Availability

The analysed data from the current study are available from the corresponding author upon reasonable request.
